# Applying risk matrices for assessing the risk of psychosocial hazards at work

**DOI:** 10.3389/fpubh.2022.965262

**Published:** 2022-09-06

**Authors:** Yacine Taibi, Yannick A. Metzler, Silja Bellingrath, Ciel A. Neuhaus, Andreas Müller

**Affiliations:** ^1^Institute of Psychology, Department of Work and Organizational Psychology, University of Duisburg-Essen, Essen, Germany; ^2^IfADo – Leibniz Research Centre for Working Environment and Human Factors, Dortmund, Germany

**Keywords:** risk evaluation, risk matrix approach, occupational stress, occupational safety, work design, mental health, occupational health

## Abstract

Although wide-ranging amendments in health and safety regulations at the European and national level oblige employers to conduct psychosocial risk assessment, it is still under debate how psychosocial hazards can be properly evaluated. For psychosocial hazards, an epidemiological, risk-oriented understanding similar to physical hazards is still missing, why most existing approaches for hazard evaluation insufficiently conceive psychosocial risk as a combination of the probability of a hazard and the severity of its consequences (harm), as found in traditional risk matrix approaches (RMA). We aim to contribute to a methodological advancement in psychosocial risk assessment by adapting the RMA from physical onto psychosocial hazards. First, we compare and rate already existing procedures of psychosocial risk evaluation regarding their ability to reliably assess and prioritize risk. Second, we construct a theoretical framework that allows the risk matrix for assessing psychosocial risk. This is done by developing different categories of harm based on psychological theories of healthy work design and classifying hazards through statistical procedures. Taking methodological and theoretical considerations into account, we propose a 3 × 3 risk matrix that scales probability and severity for psychosocial risk assessment. Odds ratios between hazards and harm can be used to statistically assess psychosocial risks. This allows for both risk evaluation and prioritizing to further conduct risk-mitigation. Our contribution advances the RMA as a framework that allows for assessing the relation between psychosocial hazards and harm disregarding which theory of work stress is applied or which tool is used for hazard identification. By this, we also contribute to further possible developments in empirical research regarding how to assess the risk of workplace stress. The risk matrix can help to understand how psychosocial hazards can be evaluated and organizations can use the approach as a guidance to establish a suitable method for psychosocial risk evaluation.

## Introduction

To ensure a safe working environment and to prevent possible accidents and health impairments, systematically assessing risk factors of the work environment is essential. Risk assessment is a systematic process to combat potential hazards at work and combines three elements: hazards, harm, and risk (1). Hazard is defined as a work characteristic that has the potential to cause harm. Harm is a possible detrimental consequence of a hazard for the health and safety of an employee. The risk is then defined as the chance that a harm will be caused by a hazard (2). Based on this approach, risk is often described as a product of probability and consequences (3), i.e., the probability that a hazard will cause harm, and the severity of that harm (4). The systematic assessment of physical hazards (e.g., toxins, noise, or heat) is nowadays an established measure in occupational safety and health (OSH) and has contributed to a significant decrease in the incidence of accidents at work in many industrialized countries (5, 6). However, profound changes in the nature of work like increasing digitalization, or the substantial rise of service work, result in an increasing importance of psychosocial hazards at work (7). Consequently, health and safety regulations require companies to also include psychosocial hazards within risk assessment (8).

Although there is robust empirical evidence that specific psychosocial work characteristics, such as low social support or high job demands, can impair the mental and physical health of employees (9–12), the question of how psychosocial hazards should be evaluated in the context of risk assessment is still under debate and the systematic assessment of psychosocial risks has so far been insufficiently implemented (13, 14). While numerous tools for hazard identification have been published in recent years, research has shown that different methods for risk evaluation produce differing results in risk assessment (15). It is surprising that this crucial fact has not been addressed more intensively by applied science, because it implies that following measures of risk-mitigation and work re-design could fail at promoting health and well-being of the workforce to an unknown extent (16) which is a core principle of OSH.

Challenges arise from the lack of comparable threshold values as reference data, or from the fact that psychosocial hazards sometimes occur only in the context of a specific combination of working conditions [e.g., high demands and low control (17)]. In addition, psychosocial hazards are often associated with multiple harms, as the effect of a hazard is often mediated indirectly via psychological processes and furthermore occurs with a time lag (18, 19). But there is also a conceptual shortfall that possibly results from the understanding of a psychosocial hazards as being determined on a population-based level (20). Many tools for hazard identification exclude the assessment of health-related variables in favor of evaluating hazards according to statistical measures of location, instead of directly associating hazard and harm. In addition, it seems unclear which health-related variables, or outcomes more in general, are best suited for this purpose because of the wide variety in the relation of psychosocial hazards and outcomes. However, it should not be forgotten that this complexity likewise applies to associations between physical work factors and health.

Against this background, we want to contribute to the methodological development of psychosocial risk analysis. In particular, we want to present an adequate calculation of risks based on psychosocial hazards as a necessary precondition to prioritize preventive steps in the risk management process. For this purpose we propose an advanced theoretical conceptualization of the risk matrix approach (RMA) as a method that can enhance the validity of risk analysis, discuss the avantages of the RMA against other established methods and propose a procedure to build a RMA for assessing psychosocial risks.

### Study objectives

The main objective of our study is to conceptually enhance the RMA for assessing the risk of psychosocial hazards. With that, our contribution combines the approach as an established and proven method for assessing risks related to physical hazards with psychological theories of healthy work design (17, 21, 22). First, we compare and rate existing procedures of psychosocial risk evaluation regarding their ability to reliably assess and prioritize risk. Second, we construct a matrix for specifically assessing psychosocial risk. To be able to use the matrix for risk evaluation, we discuss the theoretical and methodological steps necessary to scale the probability and severity of the approach. Based on our considerations, we finally present a graphical representation of a 3 × 3 risk matrix that uses odds ratio between psychosocial hazards and harms to statistically assess psychosocial risks. Using the constructed matrix, we provide recommendations for the development and application of the matrix in the organizational context and finally, we address possible challenges during RMA development.

Thereby, we aim to contribute to the development of a theoretical sound, empirically proven and practically useful assessment method for the risk assessment of psychosocial hazards. The proposed risk matrix can provide a conceptual framework for further empirical research. Organizations can use the approach as a guidance to establish a method for psychosocial risk assessment that is understandable by all stakeholders.

### Methodological approaches for the risk assessment of psychosocial hazards

Psychosocial risk assessment is a multi-stage process that includes the steps preparation, screening, action planning, implementation, and evaluation (23). In the screening phase psychosocial hazards are identified that are associated with a health risk to derive risk-reducing measures in the action-planning phase. Subsequently, the measures are implemented and evaluated regarding their effectiveness. A critical point in the process of psychosocial risk assessment is the transition from the screening to the action-planning phase. Based on screening results, it has to be decided whether the manifestation of a certain work characteristic causes a health-risk and therefore appropriate measures have to be derived. But how to assess the likelihood of a health-risk occurring from psychosocial work hazards and how results can be translated into actionable information is still scarcely researched (15, 24, 25).

For the implementation of the EU Directive on psychosocial risk assessment, international standards were published (26). The international standard proposes the framework of a stimulus-organism-reaction model and refer to the terms *mental stress* and *mental strain* (27). According to this concept factors influencing mental stress are external to the individual and the reaction to stressors take place within the individual as a strain reaction. Therefore, as part of risk analysis, working conditions needs to be assessed and adjusted. The international standards apply an ergonomic rather than an individual-centered clinical design approach. For the design of working conditions, the main advantage of this approach is that the responsibility for managing psychosocial risks is not shifted to employees, e.g., through behavioral preventive approaches for dealing with health-related problems. The aim is to design adequate working conditions and not to change employees. The approach leads to the fact that legal requirements for assessing psychosocial risks demand that working conditions affecting the employees have to be documented, but not the effects itself (28). Risk is generally calculated as the combination of hazard and harm (4). Methods for assessing psychosocial risks often do not fulfill this requirement, as the effects, e.g., in form of health-related outcomes, are not considered. For a complete risk calculation, the consideration of possible health-related effects of identified hazards are missing. For workplace design it is still important to refer to an ergonomic approach. Health problems should not be individualized to the detriment of workers, but a necessary and important parameter should be considered within the framework of risk assessment.

The RMA considers both factors when calculating risk and can thus be regarded as a suitable method. Before addressing the potential benefits of RMA for psychosocial risk assessment in more detail, we first consider the advantages and disadvantages of existing approaches. The studies (15, 24, 29) examined the following approaches, which we will look at in more detail in the next section:

a) The uniform cut-off procedure, where a uniform mainly theoretically derived scale score for all measured work characteristics is used as a cut-off value.
b) The value-based cut-off approach calculates empirical thresholds *via* receiver operating characteristic (ROC) analysis in relation to clinically approved health-related measures that differentiate between individuals who reach a critical value of a health-related outcome. The goal is to determine a risk factor threshold that represents an optimal ratio between true-positive rate and false-positive rate to predict a health impairment i.e., a harm. This allows the estimation of empirical cut-off values, for example for questionnaire data.
c) The reference value-based approach compares data with available reference values from previous risk assessments in the same company or comparable other companies. Through defined rules for the deviation of organizational results from the reference value, psychosocial work characteristics are selected for which measures must be derived.
d) The Clark and Cooper approach [CCA (30)] calculates a risk using a combination of the frequency of a hazard and the correlation between each hazard and the relevant harm.

### Discussion of the quality of existing approaches for assessing psychosocial risks

To discuss the quality of methodological approaches presented in Section “Methodological approaches for the risk assessment of psychosocial hazards”, it must be clarified that we differentiate between the instrument used to measure hazards (e.g., questionnaire) and the procedure used to assess the risk based on identified hazards (e.g., reference value-based approach). We generally require that instruments used to measure psychosocial hazards are based on established theories and fulfill methodological quality criteria like reliability, validity, and utility (31).

We assume that methodological quality of approaches to assess the risk of psychosocial hazards can be assessed using three criteria: (1) Criterion validity: A fundamental requirement for a sufficient quality of approaches to assess the risk of psychosocial hazards is that they must comply with the risk definition, defined as the chance that a harm will be caused by a hazard. For this, there must be a sufficient criterion validity of the approach to predict possible harms of psychosocial hazards. Thus, the approach must consider possible health-related effects and identifies this relationship validly. (2) Completeness: Another quality characteristic that can be used is how comprehensively health-related effects are considered. For example, does the procedure only refer to specific harms or are a wide range of possible effects considered. A comprehensive assessment of possible health outcomes is relevant, otherwise the risk may be underestimated. To be able to assess the health-risk comprehensively, a systematic evaluation of the effects across different health-related outcomes is important. (3) Clarity: The aim of a risk evaluation tool is to ensure a transparent and clear decision process, that is based on best empirical knowledge and reflects the understanding of involved stakeholders (32). For this purpose, the methodology should be able to identify the risk in a comprehensible and understandable way. Table 1 shows a summary of the approaches regarding the chosen evaluation criteria, which we explain in more detail in the following section.

a) The uniform cut-off procedure is based on the logic that for a work characteristic - not necessarily for the specific operationalization of this work characteristic - theoretically plausible and empirically proven relationships to health impairments exist and that it can therefore be assumed ex-ante that if a mean score of a work unit in a screening indicates a high level of this work characteristic, a risk factor is present. A uniform cut-off value is then set for all hazards, above which a need for action is indicated. Ergonomic measures in particular use this approach and suggest a moderate need for action in analogy to a traffic light system (1, 33, 34). It is assumed that the probability of a hazard is greater the more employees report an exposure, and a moderate frequency represents an unacceptable level of the risk. Even if the instrument used has a strong theoretical foundation, a uniform cut-off value across work characteristics is difficult to justify, as different stressors are very likely to have different threshold values for different health impairments (29). The authors state, that the scale scores are arbitrarily defined and meaningless to the actual risk for employee health impairment. Thus, the approach does not fulfill the necessary requirement of criterion validity, as it does not consider specific associations across different possible health impairments, as the same value is assumed for each work characteristic (29). Since no different health-related effects per hazard are considered, the approach does not comprehensively assess the health-risk. Notwithstanding this criticism, the use of a traffic light system makes the risk assessment understandable and comprehensible.
b) Cut-off scores are specific values of questionnaires that determine when a test result is positive or negative, e.g., the occurrence of depression. In the context of risk assessment, this value indicates whether there is a health risk for a specific scale. When developing criterion-related cut-off values for questionnaires, ROC analysis can be used to predict health-related outcomes respective harms. Empirically calculated correlations between hazards and harms are also taken into account in this approach, so satisfactory criterion validity can be assumed. The determined cut-off scores indicate a health-risk for a specific scale value and a health-related outcome.
Disadvantages of this approach are that cut-off values are only related to a specific harm (e.g., depression) and previous studies only consider a small set of possible outcomes (29, 35, 36). Thus, it is possible that no risk effect has been identified for a working condition related to health impairment A, but this does not exclude the existence of a risk effect related to health impairment B. However, in the context of risk assessment, the evaluation of risk effects that are based on single outcomes is not sufficient. To be able to assess the health-risk of psychosocial work characteristics comprehensively, a systematic evaluation of effects across different harms is important (11). It would therefore be necessary to calculate cut-off values for different hazards with a wide range of health-related outcomes. Moreover, clarity of the risk assessment may be limited because there are no rules to prioritize the selection of hazards for risk-mitigating measures when many hazards exceed the cut-off value.
c) To establish reference values, population-based median or mean values of psychosocial work characteristics are calculated across different occupational groups and/or sectors [see for example (37)]. To assess health-related effects, the concordance of values with individually reported exposures is compared. Risks are therefore identified at a population level, which are then considered in the risk assessment. For a variety of psychosocial work characteristics, the explanatory power of health-related effects due to the profession or occupation is low (38). Kroll (39) notes that the approach is suitable for physical but not for psychosocial hazards. Satisfactory criterion validity is therefore not given due to low explanatory power. In addition, it must be noted that risks determined at the population level do not necessarily apply in an organization-specific risk assessment. Reference values represent a relative assessment criterion. If an organization has lower values than a reference population that is already associated with high risks, it cannot be concluded that no risks exist in the organization.
The approach does not comprehensively assess possible health effects for certain hazards, but it can be assumed that a comparison of hazards with a representative population sample reflects the understanding of involved stakeholders, risk assessment becomes comprehensible, and the approach thus satisfies the criterion of clarity.
d) Unlike previous approaches, the CCA directly incorporates the relationships between hazards and harm, by using a combination of the frequency of a hazard and the correlation between each hazard and theoretically founded harms. This allows different risk effects of psychosocial work characteristics to be included. It can therefore be assumed that the criterion validity is sufficient, as the relationship between hazard and harm is taken into account. Furthermore, comprehensive health effects are considered, as correlations between hazards and theoretically established harms are considered.
However, the procedure does not provide thresholds when there is an obligation to derive measures. Instead, all measured work characteristics are ranked for prioritization. In this way the method does not provide a statement about the absolute health risk of a certain psychosocial work characteristic and no statement can be made about the number of acceptable questionnaire values (29). As a result, the method shows deficiencies with regard to clarity.

**Table 1:** Quality evaluation of methodological approaches to psychosocial risk assessment.

	**Criterion**	**Completeness**	**Clarity**
	**validity**		
a) Uniform cut-off procedure	–	–	+
b) Cut-off value	+	–	–
c) Reference value	–	o	+
d) Clark and Cooper approach	+	+	–

+: Fulfills the criterion completely. o: Partially meets the criterion. –: Does not fulfill the criterion.

### Risk matrix approach

**Figure 1:**
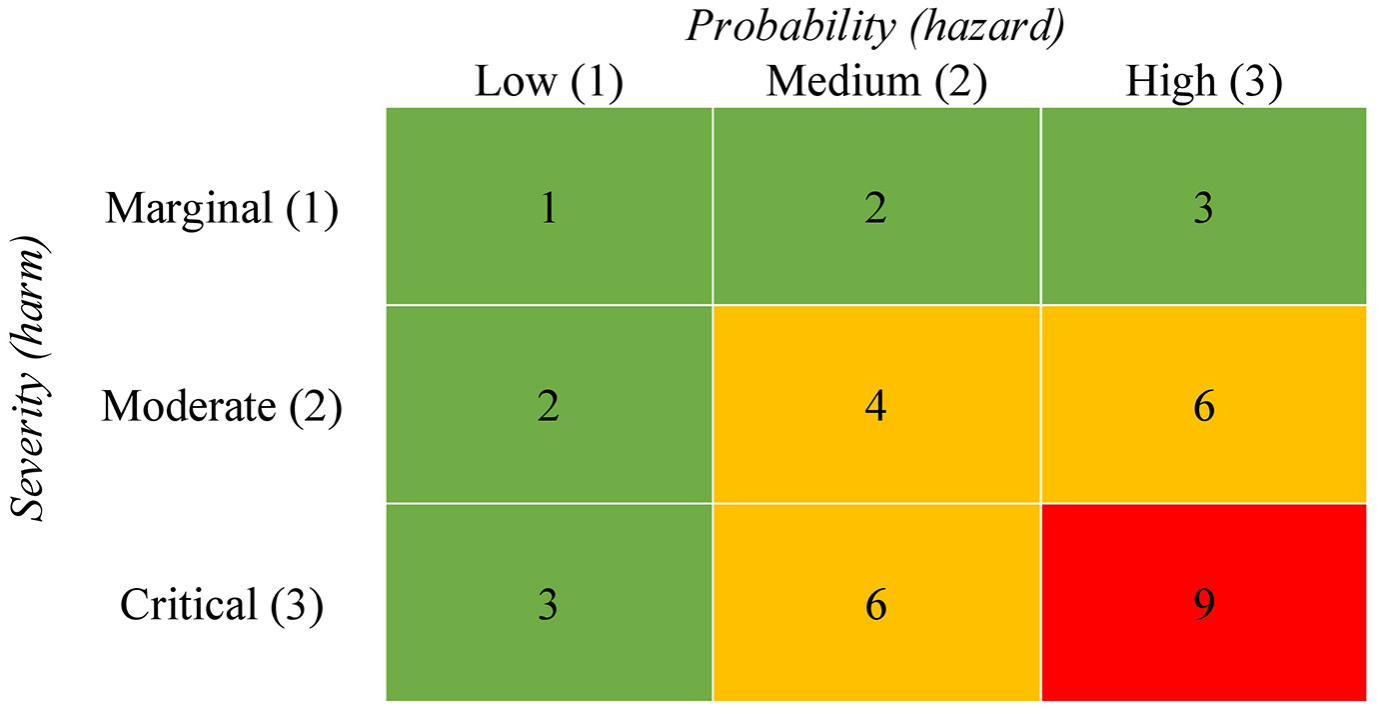
Exemplary illustration of a risk matrix. The numbers in cells quantify the risk for a harm by multiplying the two axes. The grid is divided into three different risk categories. Green indicates a low, yellow a medium and red a high risk.

Generally, risk assessment can be divided into qualitative, semi-quantitative and quantitative methods (40). While quantitative methods use numerical values to describe the extent of damage and/or frequency, qualitative methods present the results as non-numerical estimates in the form of descriptions or recommendations. The semi-quantitative method combines the procedure of the two methods. Among others, assessing physical risks by an RMA is a frequently used semi-quantitative method (41) and a traditional hazard analysis technique to specify risks. Risk matrices are widely used, and national and international standards have promoted the introduction of risk matrices (42). The approach calculates the risk as a combination of the likelihood of an adverse event (hazard) and the negative consequences (harms) (32). The matrix is often illustrated as a table that systematically contrasts different categories of probability of occurrence of a hazard in rows (or columns) with different categories of severity of harms in columns (or rows, respectively). Accordingly, each cell of the table shows a specific degree of urgency with respect to the derivation of measures [(42), see Figure 1]. The calculation process of the RMA is represented with the formula *r* = *p* × *c*, where *p* is the probability of hazard and *c* is the severity of a harm (43). To apply values to cells in the matrix, point values must be assigned to the axes. Figure 1 shows a possible representation of the approach where we have assigned the points one to three to the axes in ascending order. The risk score is obtained by multiplying the two axes. Based on the risk score, threshold values can be defined above which a need for action exists. For example, a critical harm degree (3) with the probability “always” (3) has a risk score of nine and thus a high urgency to reduce the risk with appropriate interventions. The grid is usually divided into fewer risk categories and summarized by colors, such as green, yellow, and red, to represent low, medium, and high risk (32).

### Possible advantages of the RMA

In the following section we discuss the advantages of the RMA for the assessment of psychosocial risks in respect to the evaluation criteria presented in Section “Discussion of the quality of existing approaches for assessing psychosocial risks”. As the RMA assess risk as a combination of the likelihood of an adverse event (hazard) and negative consequences (harms), the approach considers possible health-related outcomes of hazards. Thus, the definition criteria of risk are met, and sufficient criterion validity can be assumed, provided that theoretically sound and empirically validated outcomes are considered in the development of the risk matrix. In the risk assessment it is necessary to clarify which negative consequences are relevant when looking at a multitude of possible consequences (44). The selection of relevant consequences can be determined based on empirical evidence of possible health-related effects of psychosocial work characteristics. The selection of these outcomes can be assigned to the harm categories of the matrix (see Figure 1) and summarized within the categories if they remain comparable (32). Different types of severity, such as the impact on environment, human health, or economy, cannot be summarized within a category. The classification of outcomes into harm categories requires conceptual understanding and empirical validation of the severity of adverse outcomes (e.g., marginal: short-term reversible strain like fatigue, severe: chronic disease like depression). This enables risk matrix to include a broad range of different outcomes in the calculation. Thus, it can be assumed that the approach fulfills the criterion of completeness. Moreover, as the RMA enables a comparison of hazards across different severity levels, it becomes possible to make a statement about what kind of health risks are likely for a certain hazard, and thus allows a prioritization with respect to the development of measures. Furthermore, by dividing the grid into different risk levels, the risk matrix can provide threshold values above which a health risk exists, and risk-reducing measures must be derived.

Due to the intuitive graphical design of the approach and the presentation of different risk levels, the risk for specific hazards and the respective necessary instructions for action, the procedure allows for a clear and comprehensible risk assessment. This makes it well received in manufacturing, service work and other industries (40). Even though the assessment of physical hazards using the RMA is very common, we are not aware of any publication that examines the use of the RMA in the risk assessment of psychosocial risks. Before transferring the RMA approach to the assessment of psychosocial risks however, the points of criticism mentioned should be considered.

The RMA is not without limitations. Cox (42) criticizes the subjective interpretation of the input to matrices. Duijm (32) also notes, that the a priori assessments of probability and severity of adverse events are not always precise. Thus, to achieve an objectification of a risk assessment of psychosocial hazards based on the RMA, theoretically sound and empirically validated health models that that can validly explain the relationship between psychosocial work characteristics and possible health-related outcomes must be considered. A further potential disadvantage of the approach is an imprecise classification of the risk index, as the different risk levels are grouped into categories. This can lead to insufficiencies due to the complexity of assessment problems (40). The categorization also allows the same qualitative ratings to different quantitative risks, as there can be a wide range of risks within a single category (41). The disadvantage is evident when considering the risk levels in Figure 1. For example, the yellow category includes both critical and moderate harms, whose health-related effects may differ significantly, but are in the same risk category due to the calculation process. Finally, the approach neglects uncertainty in the probability and consequence assessments, which can result in errors in the decision process (32, 41, 42, 45).

In addition to the general advantages and disadvantages discussed, we want to answer the question to what benefit the RMA has compared to previously established approaches and how the points of criticism mentioned can be countered.

## Methods

In the following section we want to propose a theoretical procedure to build a RMA for assessing psychosocial risks. Markowski and Mannan (43) propose four steps that are necessary to build a risk matrix: (1) categorization and scaling of the probability of adverse events (hazard) and the severity of negative consequences (harm), (2) categorization and scaling of an output risk index, i.e., the number of possible risk categories (3) build-up risk-based rules knowledge and (4) the graphical edition of the risk matrix. For a conceptual development of the RMA for the assessment of psychosocial risks, we transfer these steps on the illustration of a 3 × 3 matrix (see **Figure 3**).

Two scenarios are conceivable for the development of RMA:

1) An organization creates and maintains a matrix based on internal available data, e.g., from employee surveys, task analyses or statistics on sick leaves. In this scenario a RMA with high ecological validity can be developed, i.e., a high validity for the specific organizational context. But a high level of methodological expertise and a large amount of time must be invested to create and maintain the RMA. In addition, the linking of different data sets can pose data privacy issues within the organization. Moreover, the available data structure could possibly be unsatisfactory (e.g., if only cross-sectional data are available).
2) Instrument developers can aim to create matrices as an aid to interpretation. The advantage is that more resources are available to create and maintain large datasets. In addition, there are no conflicts with organizational data protection regulations, so that more clinical outcomes can be recorded if necessary and the collection of data sets can be scientifically monitored. A disadvantage, however, results from organization-specific risks that may not be identified when collecting data at a population level.

### Categorization and scaling the risk matrix

**Figure 2:**
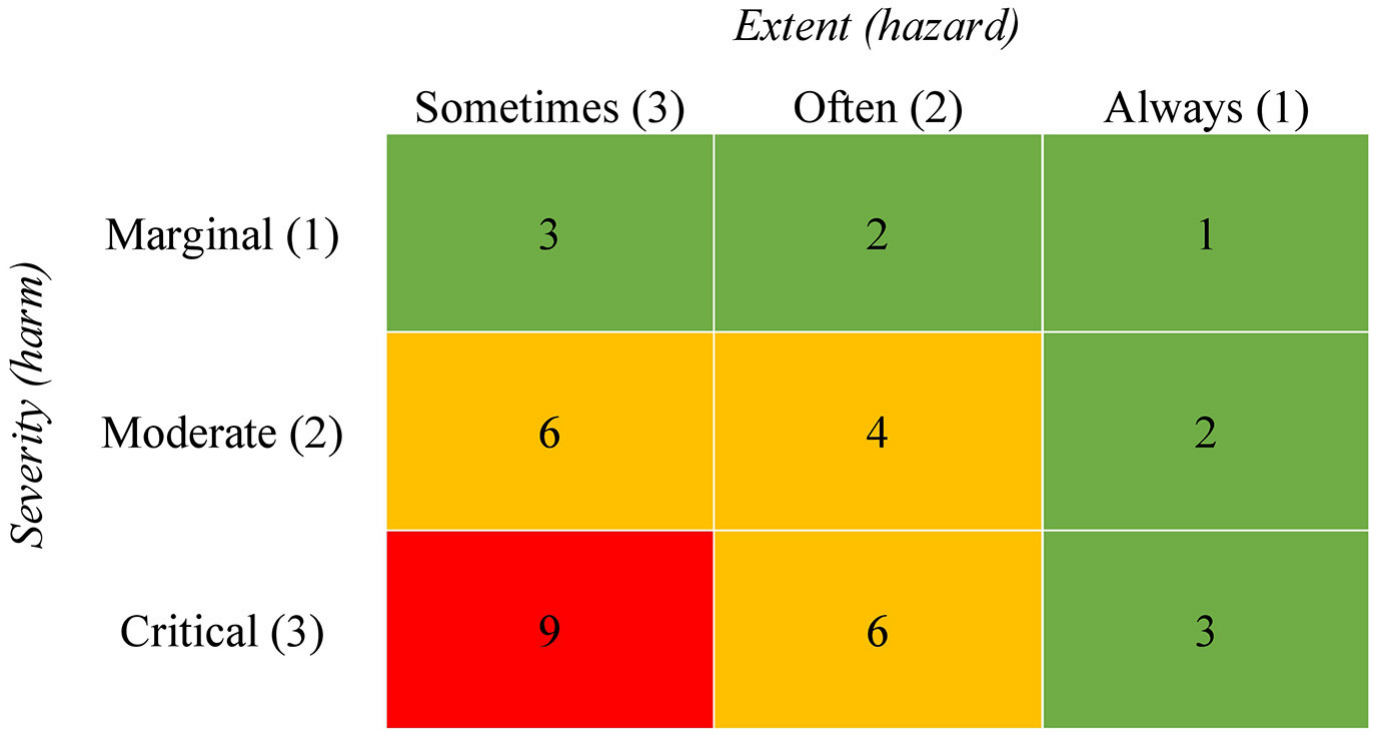
Exemplary illustration of a risk matrix with hazards as an extent score. *Note*. The numbers in cells quantify the risk for a harm by multiplying the two axes. The grid is divided into three different risk categories. Green indicates a low, yellow a medium and red a high risk.

To build the axes, the two dimensions probability (hazard) and severity (harm) needs to be scaled and categorized. The two axes must be divided into a number of categories, which finally determines the number of risk levels. Our example in Figure 2 shows three categories per axis and thus nine different risk levels. Duijm (32) note that discrete categories for the risk matrix can be identified by nominal and textual labels (e.g., high, medium, low respective never, sometimes, always). We describe the scaling process in detail in the following two sections.

First, it is important to clearly define the two dimensions of the risk matrix. In our case we define hazards as the probability of occurrence of psychosocial work characteristics that have been empirically proven to be associated with negative health consequences. Harms can be defined as the corresponding clinical and non-clinical health impairments. In order to obtain an overview of hazards and harms that need to be considered in the RMA, we focus on psychosocial aspects of work characteristics by acknowledged concepts and theories on work stress and work design. Table 2 provides an overview of relevant hazards and associated outcomes. Even though environmental or ergonomic factors (e.g., noise, temperature, etc.) may also encompass a psychosocial component, established concepts and theories on work stress and work design refer to generic psychosocial work characteristics (e.g., the dimension job demands in the job content questionnaire (51): “my job requires me to work hard”). Furthermore, these models also do not consider antecedents that cause work characteristics being classified as potential hazards. This has also been criticized for example by Parker et al. (52), when emphasizing that work design theory often fails to consider antecedes of work stress. For example, organizational design (e.g., reward system, strategy), management style or technologies used in the organization have an influence on psychosocial work characteristics and therefore on potential hazards. Antecedents of psychosocial work characteristics play an important role for the work design. However, they are initially negligible for the development of the RMA.

**Table 2:** Relevant work characteristics and key outcomes across different work-related health models.

**Model**	**Work characteristics (hazards)**	**Key outcomes (harms)**	**Study**
Job characteristics model	Skill variety Task identity Task significance	Sickness absence	(46)
Job demands–resources model	Job demands Job resources (e.g., rewards, security)	Psychological strain Burnout	(46)
Challenge-Hindrance model	Challenge demands: Workload Responsibility Hindrance demands: Role ambiguity Role conflict	Burnout	(46)
Vitamin model	Job complexity	(Emotional) exhaustion	(46)
Action regulation theory	Task variety Completeness of the work tasks	Fatigue Monotony Increased heart rate Increase in blood pressure Adrenaline release	(47)
Job demands–control model	Job demands Job control	Absenteeism (self-report and company registered) Accidents and injuries Burnout Depression Psychosomatic health complaints Psychological strain (General Health Questionnaire)	(48)
Effort-reward imbalance model	Effort: Physical load Time pressure Interruptions Reward: Esteem Security/career opportunities	Cardiovascular diseases Sickness absence (Psycho)somatic health symptoms Burnout	(49)
–	Work time control Social support	Absenteeism Musculoskeletal disorders	(11)
–	Work intensity Bullying Working hours Overtime Job strain	Depression Anxiety Psychological impairments Cardiovascular disease Diabetes	(12)
–	Destructive leadership	Affective symptoms Burnout Stress	(50)

#### Scaling the probability (hazard)

The probability axis of the risk matrix, as with other physical risk assessment methods, refers to the probability or frequency of events occurring (e.g., how often someone fell during work). Psychosocial hazards can be defined as events [e.g., Copenhagen Psychosocial Questionnaire, COPSOQ: Do you have to do overtime? (53)], but also how permanent a specific work characteristic is [e.g., COPSOQ: Is your work emotionally demanding? (53)]. In other words, usually the data refer either to the frequency or to the extent of the examined work characteristics.

The risk matrix must be interpreted differently if the *extent* of the hazard is measured compared to the *probability or frequency* of a hazard. If the extent of a hazard is measured (e.g., the level of time pressure), the risk must be classified as “high” when a hazard is associated with a critical outcome at a low level (e.g., when already a low level of time pressure is significantly associated with depression). In other words, if low level psychosocial work hazards are already linked to a critical outcome, risk-minimizing measures must be developed at low levels. Accordingly, if only high-level hazards show a relationship to health-related outcomes, this means that a health risk is only present at higher exposure levels of the hazard. In summary, this means that the content of the used instrument to evaluate the hazard has an impact on the calculation of the risk and the meaning of the cells of the risk matrix. Figures 1, 2 illustrate the classification of risk levels for the two cases described.

**Figure 3:**
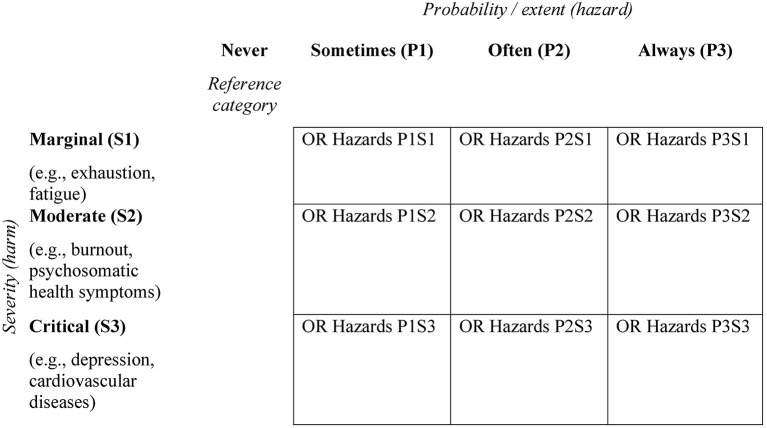
Graphical edition of the RMA for psychosocial hazards. OR, odds ratio.

For the development of the matrix, it has to be determined which categories exist to capture hazards and the results from the risk assessment can be assigned to these categories. The axis for assessing hazards must therefore be built on the data of the used instrument and be oriented to the respective scaling. Either the scale levels of the instrument can be mapped directly as categories in the matrix or classification procedures can be used to assign the results of the instrument to the hazard categories of the matrix. Notelaers et al. (54) for example recommend using the mean values of standardized questionnaires and applying statistical classification procedures. The questionnaire data can then be grouped using z-standardization. The aggregation reflects the categories of the matrix (e.g., sometimes, often, and always; see Figures 2, 3) and associations between the categories and possible health-related outcomes can be used to calculate a risk score. For our illustration, we build a 3 by 3 risk matrix with a three-level scale (sometimes, often, and always) as an example (see Figure 3).

#### Scaling the severity of consequences (harm)

To scale the dimension of harm, it is necessary to consider theoretically sound and empirically validated results between psychosocial work characteristics and health-related effects. A brief summary is provided by the most recognized concepts and theories of work stress and work design [see for example (46)]. Additional outcomes were identified in the reviews Taibi et al. (11) and Rau (12). Both studies summaries meta-analyses and systematic reviews and thus provide reliable information on possible consequences of psychosocial work characteristics.

Absenteeism and clinical health related outcomes such as depressive symptoms or nonclinical indicators of impaired wellbeing (such as exhaustion, monotony, or irritation) can be considered as outcomes, that might mediate the association between work characteristics and more severe health-impairments in terms of clinical diagnoses (55). The consideration of non-clinical indicators can be used to identify more severe health-impairments at an early stage. Empirical evidence suggests that irritation [subjectively perceived emotional and cognitive strain (56, 57)] is a preventive indicator for mental disorders (58). Consideration in the risk assessment can help prevent the development of more severe disorders based on early indicators.

In order to keep the subjective bias of the matrix as low as possible, a comprehensive consideration of possible health-related effects is necessary. Table 2 summarizes possible relevant outcomes across different work-related health models. The next step was to assign the identified outcomes to the categories of the matrix. Following Ni et al. (40) we created a 3 × 3 matrix with the degrees marginal, moderate, and critical to assess the level of harm.

For the classification of health-impairments into the three categories, we refer to the proposed time until an impairment occurs after the occurrence of a hazard. According to international norms related to mental workload (26) and work stress models such as the job demands-resources model (21, 48), we can distinguish between short-, medium-, and long-term health-impairments. A distinction can be found between synchronous stressor-strain effects that occur directly and chronic stressors that may have longer-term physical and mental effects and that take time to develop (59). Short-term health-impairments, such as fatigue, are usually not considered to be very serious and can usually be offset after normal recovery breaks or changes of work tasks (60). Even assuming adaptation effects, it is expected that psychological and psychosomatic dysfunctions usually increase with longer exposure to or stronger intensity of detrimental working conditions (61). Thus, it can be assumed that an exposure to adverse psychosocial work characteristics over a longer period of time lead to more severe health-related outcomes. This is also associated with longer incapacity to work linked to the outcome. Thus, the degrees of harm can be mapped over the duration of the development. As a result, diagnosed diseases such as musculoskeletal disorders, cardiovascular diseases, depression, or diabetes that may take longer time to develop can be classified as harms with high severity. Effects such as fatigue, monotony or increased heart rate occur directly and can be classified as marginal (26). Health-impairments that may develop in medium term, such as psychosomatic health complaints, can be assigned as a medium severity degree. Table 3 shows the selected classification for the examined health-related outcomes.

**Table 3:** Possible classification of outcomes to consequences/severity.

**Severity level**	**Outcome**
Marginal	Fatigue
	Monotony
	Increased heart rate
	Increase in blood pressure
	Adrenaline release
Moderate	Psychological strain
	(Psycho) somatic health symptoms
	Burnout
	Medium-term sickness absence
Critical	Depression
	Anxiety
	Cardiovascular disease
	Diabetes
	Musculoskeletal disorders
	Long-term sickness absence

### Calculation and graphical edition of the risk matrix

In the next section, we discuss possible calculation methods that are necessary for the conceptual development of the RMA and enable a categorization and scaling of the output risk index. For the calculation and graphical representation of the approach, we propose the procedure presented in Section “Categorization and scaling the risk matrix”:

a) Categorization and scaling hazards
b) Categorization and scaling harms
c) Empirical assessment of hazards and harms
d) Build-up risk-based rules
  d1) Binary logistic regression between “harms” and “hazards” with one degree of “hazards” as a reference category (e.g., never, see Figure 3).
    i) Calculation requires dichotomization of the “harms” into critical/non-critical values.
  d2) Statistically significant odds rations of different outcomes within a severity level are summarized.
  d3) Hazards with the calculated odds ratios are entered in the cells of the matrix.

a & b) As described in Sections “Scaling the probability (hazard)” and “ Scaling the severity of consequences (harm)”, hazards are divided into different categories depending on the scaling or classification procedure. “Harms” are assigned to severity categories based on theoretical and empirical evidence.

c) An employee survey with a standardized and validated instrument is one suitable method for empirically assessing hazards [e.g., COPSOQ (53); HSE Indicator Tool (62); Job Content Questionnaire (51)]. To be able to assess the risk as comprehensively as possible, harms in all three severity categories should also be recorded. However, not every instrument used to record hazards is suitable for this purpose, as e.g., the HSE Indicator Tool and the Job Content Questionnaire do not record health-related outcomes. The COPSOQ, on the other hand, contains scales on health-related effects such as cognitive stress or burnout that can be used to measure harms. Nevertheless, the integration of other instruments is useful for measuring clinical characteristics such as musculoskeletal disorders [The Nordic Musculoskeletal Questionnaire (63)] depression [Patient Health Questionnaire (64)] or burnout [The Maslach Burnout Inventory (65)]. In addition, company data (e.g., sick leave) can be included to consider further outcomes of the different severity categories. Biological data such as increased hart rate or adrenaline release are less suitable for the implementation in the risk assessment.

d) An appropriate way of calculating a risk score is the multiplication of the numbers which were assigned to the different categories of the axes (32). The calculated risk scores are then classified into different risk categories (e.g., low, medium, and high risk; Figure 1). However, the classification of the risk scores is influenced by a subjective assessment (40, 42). To keep the subjective input as low as possible when using the approach for assessing the psychosocial risk and to consider the particularities of psychosocial work characteristics, we have assigned health-related outcomes to the different categories of harm. How to calculate risk scores for this approach is presented in the following section:

d1) Available systematic reviews and meta-analyses provide robust empirical evidence of the risk effects of psychosocial work characteristics on negative health consequences (11, 12, 66–69). Results of these meta-analyses are usually presented in the form of correlation coefficients. The coefficients are only indirectly suitable for the derivation of risk values since risk-based statements for the evaluation of hazards assess the probability of a negative event (29).

In addition, it must be considered that organization-related data are often available in a multi-level structure and must therefore be analyzed with suitable methods. Otherwise, incorrect estimations will occur, because relationships among variables at one level do not necessarily are present at another level of the hierarchy (70, 71). Besides the classic macro-micro multi-level structure (dependent and explanatory variable are measured at a lower level), organization-related data such as sickness rates are not available for each individual, but are only reported for organizational units (e.g., per department). This results in a micro-macro structure (dependent variables are measured at a higher level and have no variance at the lower level), for which there are only a few suitable analysis methods so far (71, 72).

To make risk-based statements, dichotomous variables are necessary and thus different calculation methods. In medical research, for example, the risk of health impairments or diseases between dichotomous predictors and outcomes is calculated by odds ratios (73). The measure represents the odds that an outcome will occur given a particular exposure, compared to the odds of the outcome occurring in the absence of that exposure (74). This approach can be transferred to the calculation of the RMA if the outcomes considered are dichotomized. Dettmers and Stempel (29) consider odds ratios as a solid basis for transparent decision making and as a basis for establishing rules on how to proceed during the different stages of psychosocial risk assessment. Therefore, we propose a method that allows the calculation of odds ratios. For this purpose, a binary logistic regression between psychosocial hazards and harms can be used to predict the probability of the dichotomous outcome variable. For the calculation of the binary logistic regression, a reference category of the predictor (here a harm) must be selected. For a reasonable interpretation, either the highest or the lowest degree is suitable. In our example, we chose “never” as the reference category which refers to the degree when the probability of the psychosocial work characteristic is zero. This category can be selected for the calculation but is not used in the final graphical representation of the matrix.

d2) All significant odds ratios between one measured psychosocial hazard and all health-related outcomes within one category (see Table 3) are averaged. This score represents the average risk effect of a hazard on associated harms of a certain severity category (see Figure 3).

d3) Hazards with statistically significant odds ratios are entered in the cells. If, for example, the hazard “lack of social support” in the degree “often” shows a statistically significant association with psychosomatic health symptoms and burnout, “lack of social support” with the corresponding averaged odds ratio is entered in the cell of the column “often” and the row “critical.”

## Results

In the following section we want to propose recommendations for the development and application of the matrix in an organizational context. The proposed approach is a theoretical concept and has not yet been calculated using existing data. Our proposal is intended to support further empirical research. The prioritization of risk-mitigating measures in the risk management process using the RMA is based on the following four steps:

1. Assessing hazards with a suitable instrument (e.g., conducting an employee survey based on the COPSOQ).
2. Calculating the means of all hazards.
3. Assessing the risk of each hazard by comparing the hazard means with the risk values in the cells of the risk matrix.
4. Prioritize the development of measures according to the risk of the hazards.

For the selection of risk-mitigating measures, hazards that indicate a statistically significant odds ratio in the association with a harm are relevant, as they indicate a risk for the health outcomes of the respective harm level.

**Figure 4:**
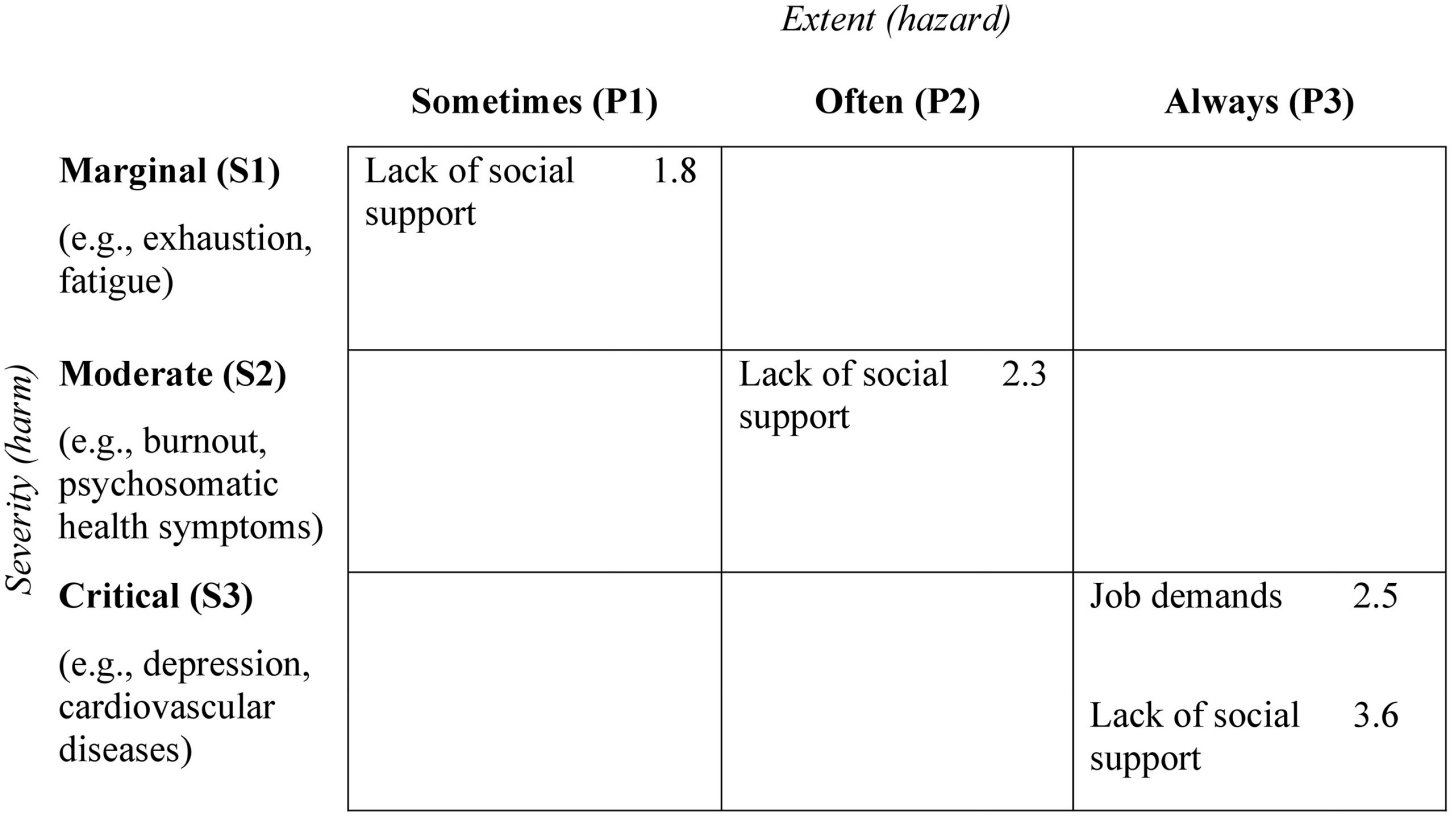
Example of a risk matrix with fictional data for the selection of risk-mitigating measures. The values represent averaged odds ratios between the measured hazards and all health-related outcomes within one category.

*Example based on* *Figure 4*: In level P3, a risk was identified for high job demands and low social support, as significant correlations to corresponding health outcomes exist. If the risk assessment identifies a value for job demands that can be assigned to the category always (P3) there is risk of critical health impairment and measures must be implemented. If the value for job demands can be assigned to categories P2 or P1, no measures are required, as no health risk can be identified. For social support a risk is identified for every extent and therefore, risk-minimizing measures must be derived for this scale from level P1 onward. However, it must be kept in mind that for category P1 only a marginal health risk is evident. Therefore, within the framework of risk prioritization, it may make sense to derive measures only at higher severity categories.

### Limitation

The proposed method for assessing the risk of psychosocial hazards has a few methodological limitations that should be accounted for. Some of the suggested health-related outcomes are captured on an abstract level and can therefore cover heterogeneous aspects. Musculoskeletal or cardiovascular diseases include medical conditions that can have varying degrees of impact on personal health. A heart attack may have more severe effects than myocarditis, but both cases are grouped under cardiovascular disorders. Therefore, studies are needed that show the relationship between psychosocial work characteristics and possible effects, that uses a fine-grained classification of health-related outcomes.

A further disadvantage arises from the fact that the RMA can be insufficient due to the complexity of assessment problems (44). The two axes of the RMA represent complex interactions in reality. Therefore, the causes for the occurrence of risks may be disregarded. Instead of a non-numeric calculation process, the authors (40) propose an arithmetic extension of the risk matrix, which we have already partially considered in our development via the calculation of odds ratios. Other methods for assessing psychosocial risks have a similar problem, as previous approaches only consider one parameter of the risk definition and ignore health-related effects. The RMA goes one step further and, in addition to hazards, also considers harms. For the practical development of the approach, it can be examined whether calculations from engineering-based approaches to risk assessment can be considered.

The performance of RMA to improve risk management decisions depends on the joint distribution of the attribute's probability and consequences. Since risk matrices are normally used when quantitative data is limited, the joint distribution is mostly uncertain (42). In order to adequately develop the RMA for the assessment of psychosocial risks, sufficient knowledge about the statistical joint distribution of probability and consequence is necessary. Whether the matrix is developed within an organization or by instrument developers, the joint distribution of probability and consequence should be checked before developing the RMA.

Another limitation arises from the necessary artificial dichotomization of health-related outcomes to calculate odds ratios. A transformation of continuous variables into dichotomous variables is accompanied by a loss of information. In addition, rules for categorizing outcomes into critical and non-critical values must be defined. In psychological research, outcomes are often measured using continuous variables. There are questionnaire procedures for the measurement of health-related outcomes that define threshold values (e.g., World Health Organization-Five Well-Being Index). In the medical context, a dichotomous classification is not uncommon, e.g., the detection of diabetes using the average blood sugar level (75). In the assessment of diseases, a distinction is made in most cases between present and absent and the degree of expression is not mapped using a continuous scale. For the calculation of the RMA, it must be considered that an operational guideline for dichotomization must be available for all included outcomes in order to be able to determine them as objectively as possible. Especially for psychological outcomes, which are increasingly available as continuous variables, thresholds must be defined.

## Discussion

Due to changes in work requirements in many occupational fields, the importance of psychosocial risk factors increases. Health and safety regulations require companies to include psychosocial hazards in the risk assessment, but the systematic assessment has so far been insufficiently implemented. Existing approaches to assess psychosocial risks do not completely assess the risk in the understanding of the definition or do not provide a clear and comprehensible risk assessment. Since approaches to the analysis of physical hazards can only be transferred to the context of psychosocial risks to a limited extent, the main objective of our study was a conceptual advancement of the RMA to enable the risk assessment of psychosocial hazards with this method. The approach is proven in relation to other (e.g., physical) risk factors, but so far, there are no risk matrices for the assessment of psychosocial risks. The assumed advantage of the RMA over other methods is that it calculates risk as a combination of the probability of an adverse event (hazard) and the negative consequences (harm). Furthermore, the method is transparent, and the graphical intuitive representation enables all participants to understand the risk assessment and prioritization for further risk-mitigating measures. To scale the probability, we classified hazards into different categories, either by transferring the scale of a used instrument onto the matrix or by applying statistical classification procedures. To scale the severity, we assigned harms into categories based on theoretical and empirical evidence. Odds ratios were calculated by logistic regression and hazards with significant results were entered into the cells of the matrix. To prioritize risk-mitigating measures, the risk is classified by comparing the values from the risk assessment with the hazards determined in the risk matrix. The RMA may provide critical values to prioritize different hazards, also regarding possible risk-mitigating measures to be derived. By designing the RMA, we contributed to the advancement of a theoretical sound, empirically proven and practically useful assessment method for the risk assessment of psychosocial work hazards. Our approach justifies the risk assessment based on a variety of empirically and theoretically founded health-related outcomes, and comprehensively includes the effects of psychosocial hazards.

It should be considered that the RMA has advantages over other methods in risk assessment of psychosocial hazards, but the development is more complex, time-consuming, and thus also more cost-intensive, as multi-layered data material and a wide range of possible health-related outcomes must be considered. Nevertheless, a well-founded risk screening is essential for the risk assessment since the selection of hazards for the derivation of risk-mitigating measures is based on these results. An insufficient screening can result in the development of measures that fail to reduce the health-endangering psychosocial work characteristics. This would miss the key objective and the health and safety of the employees cannot be guaranteed.

Two scenarios of RMA development are conceivable. First, authors developing instruments that assess psychosocial risks (51, 53, 62) may aim to create the RMA as an interpretive tool. Sufficient capacity can be created within research projects to generate large (long-term) data sets and use them for compilation. Another option is the development of a risk matrix within organizations. This results in a higher ecological validity, as the data is collected within the specific work situation. However, the development effort can be very high, especially for small and medium-sized enterprises, because either little expertise is available, or the data structure is insufficient for substantiated statements. In addition, clinically relevant outcomes may not be recorded due to company-specific data protection guidelines and the data situation is insufficient for the assessment of higher severity levels. A possibility to reduce the effort is to limit the number of included health-related outcomes. Instead of recording all proposed outcomes, only a small selection per severity degree can be considered. Before development, it can be decided which outcomes are relevant in the risk assessment and the company limits itself to a smaller selection. It should be noted that the reduction of outcomes can reduce the validity of the risk assessment. Any relationship between hazard and harm that is not mapped can lead to hazards not being identified as a health risk.

It must, however, be kept in mind that the choice of health-related outcomes can influence the results of the risk assessment. During the last decades many studies have demonstrated that specific psychosocial work characteristics can impair the mental and physical health (11, 12, 21, 66–69, 76). A key point is the role of leadership behavior. Especially destructive leadership is a factor that is negatively associated with the health of employees (50, 77, 78). Furthermore, leadership style operates as a mediator. Communicative processes like feedback or availability of information can reduce other hazards (such as role, task, and interpersonal conflicts) and contribute to the formulation of efficient problem-solving strategies (50). A destructive leadership style, on the other hand, can encourage bullying and harassment (79). Leaders are influenced by the organizational structure and culture, but leaders also have a possibility to shape and change it (78). As the interaction has a great impact on the health of all employees, leadership behavior must be considered in the risk assessment of psychosocial hazards. When assessing hazards, it may therefore be more appropriate to use instruments that refer to multifactorial models [e.g., Work Design Questionnaire (80); COPSOQ (53)]. These instruments cover a broad range of work characteristics and include individual factors such as environmental aspects or leadership, which are not captured by acknowledged concepts and theories on work stress and work design. The large number of inter-individual differences between studies in the assessment of psychosocial risks requires an epidemiological understanding similar to physical hazards, so that the question of which health-related effects certain hazards show is raised at a population-based level (1, 20, 81). The epidemiological perspective indicates risks associated with specific work characteristics at a population level without considering organizational or occupational specific effects in the risk assessment (27, 28). To verify whether specific hazards represent a health risk in the respective organizational context, it is important to consider health-related effects in the organizational risk assessment and not solely refer to epidemiological evidence. Thus, the scientific evidence of relevant hazards and related harms provides a basic framework for building up the RMA and the aspects that needs to be assessed, however it seems reasonable to use organization-specific data for the risk calculation. Such an approach, however, can only be implemented in large organizations.

One aspect in which psychological hazards differ from physical hazards is often the time interval between exposure and effect, because physical hazards usually have immediate effects, like an accident, whereas the effects of exposure to psychological hazards may remain latent for longer time (2). To account for this specificity, we assigned more severe health-related outcomes that take longer to develop across different degrees. However, long-term data are required to map time-related effects. Within the organization, only cross-sectional data are likely to be available because of the expense and company-related guidelines. Therefore, it must be considered that a time-related effect between hazard and harm cannot be determined in cross-sectional designs. Therefore, long-term data are more suitable for an optimal calculation of the approach.

In a standard risk assessment, probability of occurrence refers to the likelihood that a harm will occur. Observation instruments, workshops or questionnaire procedures should provide information on how often or to what degree psychosocial hazards occur. In our article, we point out, that the interpretation of the risk is different if the *extent* of the hazard is measured, which is often the case in the assessment of psychosocial work characteristics, compared to the probability or frequency. It should therefore be noted that the classical assessment of the matrix for categorizing the risk levels can be misleading in the context of deriving measures. One possibility for categorization can be the consideration of the strength of identified odds ratios. In order to decide what role, the level of odds ratios can play in risk categorization, it is first necessary to review the data situation.

A review of legal regulations shows that the implementation of the risk assessment of psychosocial hazards at EU level is still insufficient (82). Furthermore, the study Langenhan et al. (83) found that many organizations have practical difficulties in sufficiently understanding and incorporating psychosocial risks into strategic decision-making. The methodological guidance of the RMA can help organizations to bridge this gap. Most importantly, the RMA facilitates and provides comprehensible guidelines for decision-making that can be translated into a safety strategy. Moreover, establishing the method can help to build a psychosocial safety climate (PSC). PSC is defined as policies, practices, and procedures for the protection of worker psychological health and safety (84). The theory assumes that psychological aspects are more important than productivity demands and that the management acts in the interest of employees' mental health (85). To accomplish a PSC, measures to improve working conditions and mental health should be implemented in a coordinated approach that includes organizational communication, management involvement and commitment (86). By establishing a suitable, standardized, and comprehensible method for psychosocial risk assessment, management demonstrates that psychosocial risks and the health of employees are considered and thereby promotes the PSC. When developing and implementing the RMA, it should be ensured that data protection is observed when using health data, that the development and application of the RMA is transparent for all employees and that the prioritization of risk-mitigating measures based on the results is comprehensible.

### Future research directions

For future research, it is necessary to calculate the RMA based on the conceptual development presented with available risk assessment data, either at the population-based level or in specific organizational settings. In the second case, objective data such as sick leave or accidents rates, which indicate serious harm and have a strong economic impact, are interesting.

To discuss the quality of existing approaches to psychosocial risk assessment, further methodological comparisons need to be conducted. For this purpose, the RMA and additional methods can be compared regarding their prognostic validity. The validity of the risk assessment can be determined by examining the extent to which psychosocial risks measured at baseline predict health-related outcomes measured after different time frames. Risk can be calculated between hazards and clinical health-related or non-clinical indicators of impaired well-being. Organizational data such as absenteeism or accident rates can be considered as outcomes.

Finally, the RMA can be evaluated in terms of utility and costs effectiveness in order to answer the question of how well the procedure can be implemented in organizations. It would be conceivable, for example, to conduct expert interviews with occupational safety and health decision-makers to be able to assess practicability or economic aspects such as costs. Another important aspect is the acceptance of the approach by employees, which can be assessed by means of large-scale employee surveys.

## Conclusion

How psychosocial hazards should be properly evaluated is still under debate and most existing approaches do not sufficiently assess the risk, as health-related effects are not considered. The RMA can be regarded as a suitable method for psychosocial risk assessment, as it calculates risk as a combination of the likelihood of an adverse event (hazard) and the negative consequences (harms). In our study, we were able to present the theoretical and methodological steps necessary to realize the approach for psychosocial risk assessment. This is done by developing different categories of harm based on psychological theories of healthy work design and classifying hazards through statistical procedures. Our contribution advances the RMA as a framework that allows for assessing the relation between psychosocial hazards and harm disregarding which theory of work stress is applied or which tool is used for hazard identification.

The proposed risk matrix can provide a conceptual framework for further empirical research and help to understand how psychosocial hazards can be evaluated validly in the context of risk assessment. Organizations and researchers can use this guidance to establish the RMA and thus provide a valid method to assess the risk of psychosocial hazards that is understandable by all stakeholders and provides clear decision-making that can be translated into a safety strategy.

## Data availability statement

The original contributions presented in the study are included in the article/supplementary material, further inquiries can be directed to the corresponding author/s.

## Author contributions

YT, YM, and AM formulated the research goals and developed the methodology. YT wrote the original draft and contributed to manuscript revision, editing, and visualization. YT, YM, AM, SB, and CN drafted the article and revised it critically for important intellectual content. All authors final approved the version of the submitted manuscript.

## Funding

We acknowledge support by the Open Access Publication Fund of the University of Duisburg-Essen.

## Conflict of interest

Author YT partly employed in a company within his doctoral thesis at the University of Duisburg-Essen and is involved in the risk assessment of psychosocial hazards as part of this employment. Author YM was employed as an attending occupational health psychologist within the same company and was part of the University of Duisburg-Essen and will move to the Leibniz Research Center for Working Environment and Human Factors in July 2022. The remaining authors declare that the research was conducted in the absence of any commercial or financial relationships that could be construed as a potential conflict of interest.

## Publisher's note

All claims expressed in this article are solely those of the authors and do not necessarily represent those of their affiliated organizations, or those of the publisher, the editors and the reviewers. Any product that may be evaluated in this article, or claim that may be made by its manufacturer, is not guaranteed or endorsed by the publisher.

## Abbreviations

RMA: risk matrix approach
PSC: psychosocial safety climate
COPSOQ: Copenhagen Psychosocial Questionnaire
CCA: Clark and Cooper approach.
